# Fostering Employee Trust via Effective Supervisory Communication during the COVID-19 Pandemic: Through the Lens of Motivating Language Theory

**DOI:** 10.1177/23294884211020491

**Published:** 2022-04

**Authors:** Linjuan Rita Men, Yufan Sunny Qin, Jie Jin

**Affiliations:** 1 University of Florida, Gainesville, USA

**Keywords:** supervisory communication, motivating language theory, employee trust, psychological need satisfaction, COVID-19

## Abstract

This study examines how supervisory leadership communication during the COVID-19 pandemic fostered employee trust through the lens of motivating language theory. Drawing insight from self-determination theory, this study also reveals the mediating effects of employees’ psychological need satisfaction for competence and relatedness in this process, which help explain how supervisory leadership communication influences employee trust. Through an online survey of 393 full-time employees from various organizations in the U.S., results showed that supervisory use of meaning-making (0.15), empathetic (0.60), and direction-giving language (0.27) during the pandemic all showed significant positive effects on employee trust toward leadership and the organization directly, and indirectly through satisfying employees’ psychological need for competence and relatedness. Theoretical and practical implications of the findings are discussed.

Employees have long been recognized as the organization’s number one stakeholder. Maintaining a trusting relationship with employees is regarded as one of the core tasks for leaders and communication managers (Men, 2014; Sendjaya & Pekerti, 2010). The importance of employee trust has become increasingly evident throughout the COVID-19 pandemic. The public health crisis not only altered how businesses operate (Bersin, 2020), but also cast challenges for organizational leaders to maintain trusting relationships with employees as employee work routines were disrupted and faced enormous uncertainty.

Previous studies have demonstrated that various leadership attributes can influence employee trust at work, such as transformational (Braun et al., 2013), authentic (Wang & Hsieh, 2013), and servant leadership (Thelen, 2020), as well as leaders’ communication styles and channels (e.g., Men, 2014, 2015). In internal communication literature, leadership communication has been widely recognized as an important factor that affects employee attitude and behavior given supervisory day-to-day interactions and communications with employees (Van Quaquebeke & Felps, 2018). Supervisors are arguably the most trustworthy source of information for employees (Legood et al., 2016; Men & Bowen, 2017; Whitener et al., 1998). During turbulent such as the COVID-19 pandemic, the role of supervisory communication is perceived to be even more crucial as employees expect to hear from their direct managers information that can ease concerns about uncertainties (Skiba & Wildman, 2019) and receive clear guidance to help them to navigate disrupted work routines. Recent research has demonstrated that supervisor support during the pandemic helps reduce perceived uncertainties of employees and mitigate emotional exhaustion (Charoensukmongkol & Phungsoonthorn, 2020). However, *how* supervisors communicate plays a role out in the development of employee trust during turbulent times such as the COVID-19 pandemic, and therefore potentially amidst future pandemics remains unknown. This research gap and uniqueness of COVID-19 as a study context both necessitate the current research.

Specifically, this study intends to examine the impact of supervisory leadership communication on employee trust toward the leader and the organization during the COVID-19 pandemic through the theoretical lens of motivating language theory (MLT). As a leadership communication theory, MLT has been widely tested in various organizational and sociocultural contexts (Mayfield & Mayfield, 2018), MLT argues that leadership’s use of meaning-making, empathetic, and direction-giving language (Mayfield & Mayfield, 2018; Mayfield et al., 1995) leads to positive employee outcomes. To further examine how exactly leader use of MLT influences employee trust, the study incorporates employee psychological need satisfaction stemming from self-determination theory (SDT) (Deci & Ryan, 2000) as the potential mediator that delineates the process.

Taken together, this study predicts that supervisors (i.e., leadership) use of motivating language influences employee trust during the pandemic via satisfying employees’ psychological need for competence and relatedness. The predictive model was tested via an online survey with adult workers in the US during the pandemic in 2020. The findings provide important implications for public relations and communication management scholars, communication practitioners and organizational leaders. Theoretically, the study advances the leadership communication literature by demonstrating the potential influence of supervisory communication on employee trust during a pandemic. The study also expands the explanatory power of MLT to a unique public health crisis communication context and showcases how the theory works to influence employee trust given a specific circumstance, that is, during the COVID-19 pandemic. Practically, this study provides communication practitioners insight on how to cultivate employee trust toward leadership and the organization during the challenging, turbulent, and uncertain times of a pandemic.

## Literature Review

This study examines the influence of supervisory leadership communication on employee trust during the COVID-19 pandemic. The role of trust has been widely examined within management, organizational, and communication literature (Sendjaya & Pekerti, 2010; Six & Sorge, 2008). However, as a socially constructed phenomenon, there seems to be no unified definition of trust (Atkinson & Butcher, 2003). Generally, scholars have examined trust from three perspectives: as a trait, a process, or an emergent state (Burke et al., 2007). In the current study, we view employee trust as an emergent state that results from effective supervisory communication (i.e., supervisory use of motivating language) and employee psychological need satisfaction.

### Conceptualizing Employee Trust

Employees are likely to develop trust or distrust through their interactions with coworkers and leaders as well as their experiences with and beliefs about beneficial actions and proper policies of the organization (Burke et al., 2007; Saruhan, 2013). This reflects the dual nature of trust, which can exist both at the interpersonal level and the systems level (Straiter, 2005). In a recognition of the importance of trust toward both referents, the current study is concerned particularly with both trust in leadership and the organization.

#### Trust in leadership

Trust in leadership can be readily seen in organizational life. Particularly, trust enables employees to be vulnerable and take risks in the workplace (Whitener et al., 1998), and this assumption of risk is based on the individual’s previous positive expectations or beliefs about the behaviors and intentions of others (Rousseau et al., 1998). Furthermore, trust involves one’s willingness to rely on the other party in a given situation. Following the most frequently cited deﬁnition of trust in leadership, the current study defines this term as willingness to be vulnerable to words, actions, and decisions of his or her supervisor, regardless of the ability to control the other party (Mayer et al., 1995; McAllister, 1995; Williams, 2001). Given the inherent power structure within the organization, it is likely that employees will be subjective to influence and control from organizational authorities, which makes trust in leadership a necessary approach to enhance positive employee outcomes (Yang & Mossholder, 2010). As an essential psychological state in achieving leadership effectiveness, trust stems from two distinctive psychological processes: cognitive and affective trust (Dirks & Ferrin, 2002; McAllister, 1995). Specifically, cognitive trust focuses on available knowledge of the leader and affective trust consists of the emotional bonds between employees and the leader (McAllister, 1995).

#### Trust in the organization

Scholars have identified that trust in leadership and organizational trust are two related but distinct variables. While trust in leadership is more associated with ability, benevolence, and integrity, trust in the organization is associated with proper structures (e.g., policies and contracts), organizational justice, support, communication, and culture (DeConinck, 2010; Joseph & Winston, 2005; Men & Bowen, 2017; Tan & Tan, 2000). Gilbert and Tang (1998) determined organizational trust is related to the belief that the organization will be candid and keep commitments. According to Tan and Tan (2000), trust in organizations refers to employee confidence that the organization’s activities will be beneficial at best, or at least not harmful. Chathoth et al. (2007) indicated that trust results when competence, integrity, and openness exist within organizational relationships and actions. Similarly, public relations scholars define trust in organizations as “one party’s level of confidence in and willingness to open oneself to the other party” (Hon & Grunig, 1999, p. 19). They have identified three dimensions to trust in organizations: (1) competence: the belief that the organization can act as it says; (2) integrity: the belief that an organization is just and fair; and (3) dependability: the belief that the organization will be responsible for their words and actions (Hon & Grunig, 1999). The current study adopts Hon and Grunig’s (1999) definition of organizational trust associated with employee perception of the organization’s competence, dependability, and integrity. Employee organizational trust has been associated with various beneficial outcomes for the organization, such as employee job satisfaction (Lee et al., 2013), employee openness to change (Neill et al., 2019), reduced turnover intention (Kath et al., 2010), organizational commitment (Laschinger et al., 2001), and organizational performance (Paliszkiewicz & Koohang, 2013).

### Supervisory Leadership Communication and Employee Trust

Given the importance of employee trust for organizational effectiveness, antecedents of trust have been extensively examined. Prior research has found organizational support (Dursun, 2015), perceived organizational justice (DeConinck, 2010), leadership styles (Jung & Avolio, 2000), and leadership communication (Porumbescu et al., 2013) can all influence employee trust. However, while prior research has confirmed the crucial role of leadership in developing and sustaining employee trust (Jung & Avolio, 2000; Tuan, 2012), previous studies predominantly focus on leadership style or behaviors. For example, authentic leadership characterized by integrity, commitment to core values, objectivity in making decisions and relational transparency were found to promote employee trust (Hassan & Ahmed, 2011). Although communication is recognized as a key component of leadership (Fairhurst & Connaughton, 2014; Luthra & Dahiya, 2015), only a handful of scholars have examined how leadership communication influences employee trust (Men, 2015, Thelen, 2020). Furthermore, to the authors’ knowledge, no empirical studies have explored the role of leadership communication in fostering employee trust particularly during a global pandemic.

Supervisory leadership communication represents a major component of an organization’s internal communication system (Men & Bowen, 2017). Findings from previous research suggest that supervisory communication styles, channels, skills, and effectiveness can influence employee attitudes, behaviors, emotional capital, and organizational performance (Cardon et al., 2019; De Vries et al., 2010; Men, 2014)), including employee trust (Burke et al., 2007). To fill the aforementioned research gap and explore the impact of supervisory leadership communication on employee trust during the pandemic, this study incorporates motivating language theory (MLT).

#### MLT

Motivating language theory (MLT) provides a sound framework for understanding how leadership’s daily communication with followers works to motivate employees (Mayfield et al., 1995; Sullivan, 1988). MLT is assumed to cover most leader-to-follower speech acts, namely meaning-making, empathetic, and direction-giving language (Mayfield & Mayfield, 2009). In essence, MLT represents a dyadic communication process that needs leaders to walk the talk to motivate employees. Specifically, meaning-making language is used by a supervisor to assist employees in interpreting organizational culture and values (Mayfield et al., 2015). Empathetic language is used when a supervisor communicates consideration about an employee’s emotional needs through expressing appreciation, care, sharing feelings, and validation (Mayfield et al., 1995; Sullivan, 1988). Direction-giving language refers to a leader’s language that aims to reduce uncertainty by clarifying purposes, tasks, and reward contingencies (Mayfield & Mayfield, 2009).

Previous studies show that supervisory use of motivating language (all three forms) has positive effects on critical organizational outcomes such as employee job satisfaction (Sharbrough et al., 2006), self-efficacy (Mayfield & Mayfield, 2012), organizational citizenship behavior (Gutierrez-Wirsching et al., 2015), and organizational performance (Mayfield et al., 2015). Extending previous research, the current study intends to expand the explanatory power of MLT by examining its impact at the supervisory communication level on employee trust during the COVID-19 pandemic.

#### MLT and employee trust

Prior research has suggested MLT is a strategic communication framework that can guide leadership communication for a given situation (Mayfield & Mayfield, 2002; Sullivan, 1988). The role of leadership communication practices in fostering and maintaining trust is more critical during the COVID-19 pandemic when organizations face harsh economic realities and employee trust is eroded by abrupt changes and enormous uncertainties. In times of crisis, employees often experience fear, anxiety, frustration, and discomfort; thus, they may be in need of more supervisory guidance, emotional support, and encouragement as well as transparent, timely and frequent communication (Dirani et al., 2020). As such, a leadership approach that is responsive to the needs of employees is critical for organizations to survive the crisis (Burke et al., 2007). As noted by Stoller (2020), in response to a crisis such as the COVID-19 pandemic, motivating leadership communication is a critical part of connecting team efforts, achieving shared vision, improving employee psychological safety, and finally helping the organization to navigate through the pandemic. In particular, when leaders use direction-giving language to clarify employee job roles, changes, and task expectations during the pandemic, employees may trust leadership competence in responding to the crisis (Hackman, 2002). Leadership’s clear directions and guidance for employees at work can also reduce feelings of uncertainty which in turn may enhance employee organizational trust (Gutierrez-Wirsching et al., 2015).

Additionally, a sense of trust in leaders can be developed when leaders use empathetic language to encourage and praise workers and care for their well-being (Sarros et al., 2014). Such leadership support, care, and encouragement are much needed especially during a challenging pandemic situation (Hofmeyer et al., 2020). The emotional bond employees form with leaders may be instrumental in engendering employee trust towards the organization, as, often, leaders are messengers for the organization (De Vries et al., 2010). Leadership use of meaning-making language that empathizes value-based communication and connects employees’ personal goals with the higher purpose of the organization is inspiring and motivating, which can also bolster employee trust toward the leader (Seppänen et al., 2014). Furthermore, such language can help build shared identity inside the organization (Mayfield & Mayfield, 2018) which can foster employee trust toward the organization.

Therefore, based on the above literature, the following hypotheses are proposed:

H1: Leaders’ use of meaning-making language positively influences employee trust
toward the leader and the organization.
H2: Leaders’ use of empathetic language positively influences employee trust toward
the leader and the organization.
H3: Leaders’ use of direction-giving language positively influences employee trust toward the leader and the organization.

### The Mediator: Employees’ Psychological Need Satisfaction

To further explain how leaders’ use of motivating language influences employee trust, the study incorporated employee psychological need satisfaction as a potential mediator in this process. According to self-determination theory (SDT), psychological needs must be satisfied to maintain an individual’s integrity, mental health, and growth. Three basic psychological needs have been postulated: the need for autonomy, competence, and relatedness (Deci & Ryan, 2000). The need for autonomy refers to the desire to obtain a sense of ownership and make personal decisions without being affected by external forces (Deci & Ryan, 2000; Van den Broeck et al., 2008). The need for competence refers to feelings of self-efficacy and being skillful in activities (White, 1959). In the workplace setting, competence represents an employee feeling capable of accomplishing challenging tasks and obtaining desired outcomes (Deci et al., 2001; White, 1959). The need for relatedness is defined as the desire to maintain intimate relationships and have a sense of mutual respect and care with others (Baumeister & Leary, 1995). Employee need for relatedness is more likely to be fulfilled when he or she feels belongingness to the team and is free to communicate work-related and personal concerns (Van den Broeck et al., 2008). SDT posits that work climates that fulfill the three basic psychological needs can facilitate work engagement and motivation (Deci et al., 2001). Many empirical studies have demonstrated positive organizational outcomes of satisfying employee basic psychological needs (Deci & Ryan, 2000), such as job satisfaction (Andreassen et al., 2010; Ilardi et al., 1993), organization commitment (Lian et al., 2012; Vansteenkiste et al., 2007), work motivation (Richer et al., 2002), work performance (Baard et al., 2004), and mental health (Lynch et al., 2005).

Following the SDT, this study proposes that leadership’s use of motivating language can be influential in satisfying employee psychological needs, particularly for competence and relatedness during the pandemic. Specifically, first, leaders’ use of meaning-making language emphasizes organizational values and aligns the personal goals of employees with those of the organization. The shared values and purpose may bind employees together and enhance employee connection with the organization, thus satisfying the psychological need for relatedness (Mayfield & Mayfield, 2018; Reiche et al., 2017). Second, leaders’ use of empathetic language sends message of empathy, compassion, care, and support (Mayfield & Mayfield, 2018), which makes employees feel they are not alone facing various challenges during the pandemic. Such explicit supervisory support can also satisfy the aforementioned need for relatedness (Ryan & Deci, 2000). In addition, use of empathetic language by leadership can encourage employees to cope with challenges at work during the pandemic. When employees feel their input is respected and recognized by leadership, they may be more motivated and feel a stronger sense of competence at work (Neves & Eisenberger, 2014; Weinstein & Ryan, 2010). Third, leaders’s use of direction-giving language can help reduce role ambiguity and clarify work expectations and directions (Mayfield & Mayfield, 2012; 2017; Mayfield et al., 2015). A reduced sense of uncertainty and an enhanced sense of control can increase employee self-efficacy at work and satisfy their psychological need for competence (Fawkes, 2015). Direction giving language also includes providing employees feedback and mentoring. With clear goals depicted, employees are aligned towards the same future direction on which employees are working toward together. In this sense, leaders’ use of direction-giving language could also satisfy employee psychological need for relatedness (Binyamin & Brender-Ilan, 2018; Mayfield & Mayfield, 2012; 2018). Therefore, this study proposes the following hypotheses:

H4: Leaders’ use of meaning-making language positively influences employee psychological need satisfaction for relatedness.
H5: Leaders’ use of empathetic language positively influences employees psychological need satisfaction for competence (a) and relatedness (b).
H6: Leaders’ use of direction-giving language positively influences employee psychological need satisfaction for competence (a) and relatedness (b).

An employee’s psychological need satisfaction has been associated with their perceived relationship with the organization. Previous studies have shown that when employees feel their psychological needs are fulfilled in the workplace, they tend to develop positive attitudes and behavioral intentions toward the organization, such as a higher level of job satisfaction (Van den Broeck et al., 2016), organizational commitment (Dursun, 2015), and identification with the organization (Smidts et al., 2001). As a result of recognizing the organization’s efforts in satisfying employee needs, a trusting relationship between the organization and the employee can be further strengthened (Hon & Grunig, 1999; Tao et al., 2018). Moreover, several empirical studies in management have demonstrated that employees who report stronger levels of relatedness with the organization develop greater trusting attitudes toward the organization as well as the corporate management (e.g., Deci et al., 2017; Neves & Eisenberger, 2014). Considering the context of a pandemic, satisfying the psychological needs of employees, especially the need for competence and relatedness, can be instrumental for employees to cope with psychological and emotional challenges at work and enhance their sense of well-being (Bersin, 2020; Brown & Roloff, 2015). Psychological need satisfaction of employees is an important basis for their relationship cultivation with the organization (Tao et al., 2018). Employees are more likely to develop a trusting relationship with their leader and organization when they perceive competence by the organization in dealing with the challenges (Pfeffer, 2018; Wright & Huang, 2012). Therefore, the following hypotheses are put forth.

H7: Employee psychological need satisfaction for competence positively influences trust toward the leader and the organization.
H8: Employee psychological need satisfaction for relatedness positively influences trust toward the leader and the organization.

This study proposes employee psychological need satisfaction as the potential mediator underlying the relationship between leaders’ use of motivating language and employee trust toward the leader and the organization. Specifically, leadership’s reinforcement of organizational values and purposes, understanding subordinate needs and providing support, providing clear work instruction and meaningful feedback, may all predict to what extent an employee’s psychological needs may be fulfilled at work (Deci & Ryan, 1985). Several recent studies have also shown that such need satisfaction may inspire positive organizational outcomes, including employee trust (e.g., Deci et al., 2017; Mayfield & Mayfield, 2018; Van den Broeck et al., 2016). Likewise, we argue that during the COVID-19 pandemic, leaders’ use of motivating language can satisfy employee population’s psychological need for competence and relatedness, which in turn, may enhance employee trust toward the leader and the organization. We then propose the following hypothesis.

H9: Employee psychological need satisfaction for competence and relatedness mediate the effects of leaders’ use of meaning-making, empathetic, and direction-giving language on employee trust toward the leader and the organization.

**Figure 1.**
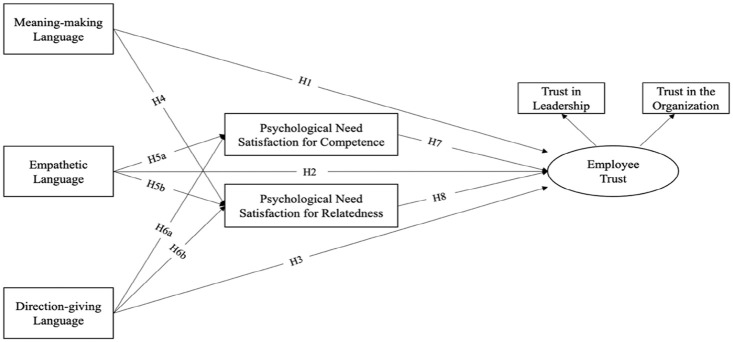
The hypothesized model of supervisory leadership communication, employees’ psychological need satisfaction, and employee trust.

In view of the preceding discussion on leaders’ use of motivating language, and employee psychological need satisfaction in association with employee trust toward the leader and the organization, the hypothesized model is presented in Figure 1.

## Method

An online survey was conducted to test the proposed model in May, 2020. The respondents were composed of employees who are currently employed full-time with an organization across different industries in the U.S. Respondents were recruited via Amazon Mechanical Turk (MTurk), which has been utilized for many social science studies for sample recruitment, and data quality confirmed in previous studies (e.g., Buhrmester et al., 2011; Sheehan, 2018). After data cleaning, a total of 393 qualified and valid responses were used for data analysis.

The average age of the respondents was 33.26 (*SD* = 9.49), and 64% of the respondents (*n* = 252) were males. More than half of respondents (*n* = 228, 58.3%) had been working in their organization for more than 5 years. In terms of the level of employment position, 42.7% (*n* = 168) were at middle-level management, followed by non-management (*n* = 135, 34.6%), lower-level management (*n* = 74, 18.8%), and upper-level management (*n* = 15, 3.8%). The majority of respondents (*n* = 346, 88%) received 2-year college degrees or above. Information technology (*n* = 102, 26.0%), manufacturing (*n* = 47, 12.0%), and healthcare and social assistance (*n* = 35, 8.9%) accounted for the top three industries in which participants were employed. Regarding the organization size, 28.5% of respondents (*n* = 112) worked in smaller organizations with fewer than 100 people, 43% (*n* = 169) in medium organizations with 100 to 499 people, and 28.5% (*n* = 112) in large organizations with more than 500 people. In addition, most of the respondents (*n* = 321, 81.7%) were working from home during the COVID-19 pandemic.

### Measures

All measurement items were drawn from previous literature and adapted to fit in the pandemic context. Seven-point Likert scales ranging from 1 (strongly disagree) to 7 (strongly agree) were used to measure focal variables. Specifically, leadership’s use of motivating language was measured by 20 items adopted from Mayfield & Mayfield (2017) to assess the use of direction-giving language (e.g., “My supervisor gives me clear directions of what needs to be done in my work during the pandemic,” α = .88), empathetic language (e.g., “My supervisor gives me praise for my good work during the pandemic,” α = .94), and meaning-making language (“My supervisor connects the work that I do to a higher purpose during the pandemic,” α = .89). Twelve items adopted from Van den Broeck et al. (2010) were used to measure employee psychological need satisfaction, including their need for competence (e.g., “I feel adequately prepared to perform my job during the pandemic,” α = .89), and need for relatedness (e.g., “I feel connected with other people in my organization during the pandemic,” α = .80). To measure employee trust, seven items adapted from McAllister (1995) were used to assess employee trust toward the leader (e.g., “I can freely share my ideas, feelings, and hopes with my supervisor during the pandemic,” α = .85), and six items from Hon and Grunig (1999) were adopted to measure employee trust toward their organization (e.g., “My organization treats people like me fairly and justly during the pandemic,” α = .92). Descriptive statistics and structural modeling analysis were used to analyze the data.

## Results

Results of the preliminary descriptive analysis as presented in Table 1 showed that respondents’ direct managers on average used moderate meaning-making language (*M* = 4.38, *SD* = 1.35) and slightly more of empathetic language (*M* = 5.02, *SD* = 1.24) and direction-giving language (*M* = 5.09, *SD* = 1.16) in communicating with followers during the COVID-19 pandemic. Respondents overall reported slightly high levels of psychological need satisfaction for competence (*M* = 5.41, *SD* = 1.00) and relatedness (*M* = 4.72, *SD* = 1.04) at work during the pandemic. They also reported slightly high levels of trust toward their direct managers (*M* = 4.91, *SD* = 1.24) and the organization (*M* = 4.92, *SD* = 1.24). Regression analysis was conducted to examine the effects socio-demographic variables on the focal variables of the study. Results showed that an employee’s level of employment position in the organization showed significant positive effects on employee trust toward the leader (β = .22, *t* = 3.78, *p* < .001) and the organization (β = .15, *t* = 2.55, *p* < .01). Therefore, position level was controlled in the follow-up SEM analysis.

**Table 1.** Means, Standard Deviations, and Correlations.

	*M*	*SD*	1	2	3	4	5	6	7	8
1. Meaning-making language	4.38	1.35	—							
2. Empathetic language	5.02	1.24	.66**	—						
3. Direction-giving language	5.09	1.16	.61**	.72**	—					
4. Psychological need satisfaction for competence	5.41	1.00	.29**	.49**	.51**	—				
5. Psychological need satisfaction for relatedness	4.72	1.04	.44**	.54**	.52**	.53**	—			
6. Trust in leader	4.91	1.24	.59**	.79**	.70**	.50**	.58**	—		
7. Trust in organization	4.92	1.24	.60**	.72**	.65**	.49**	.54**	.74**	—	
8. Position level^a^	2.16	.95	.31**	.18**	.10*	–.04	.12*	.15**	.12*	—

a Position level was measured using a four-level category system with 1 = non-management, 2 = lower-level management, 3 = middle-level management, 4 = top management.

* *p* < .05. **p < .01.

**Figure 2.**
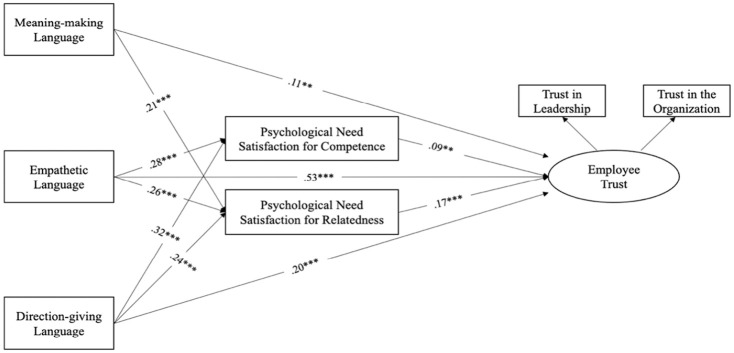
Results of the hypothesized model. *Note*. **p < .01, ***p < .001.

A two-step structural equation modeling analysis was conducted to test the hypothesized model using AMOS 26.^1^ The test of the confirmatory factor analysis (CFA) model revealed excellent fit to the data: χ^2^(4) = 8.74, *p* = .07, χ^2^/df = 2.18, RMSEA = 0.05 (90% confidence interval: 0.00–0.10), SRMR = 0.01, TLI = 0.98, and CFI = 0.99, and the standardized factor loadings from leadership trust and organizational trust to employee trust were 0.89 and 0.83 respectively, suggesting a good construct validity for employee trust. A second step evaluation of the structural model with position level controlled also yielded excellent fit to the data: χ^2^(6) = 12.09, *p* = .06, χ^2^/df = 2.01, RMSEA = 0.05 (90% confidence interval: 0.00–0.09), SRMR = 0.02, TLI = 0.98, and CFI = 0.99, and was thus retained as the final model. All the hypothesized structural paths demonstrated significant results at the *p* < .001 or *p* < .01 level (see Figure 2).

### Hypothesis Testing

Hypotheses 1 to 3 predicted the positive effects of leaders’ use of meaning-making (H1), empathetic (H2) and direction-giving language (H3) during the pandemic on employees’ trust toward the leader and the organization (see Table 2). Results provided support for all three hypotheses. Specifically, leaders’ use of meaning-making language showed a small positive effect on employee trust toward the leader and the organization (β = .11, *p* < .01). Leaders’ use of empathetic language demonstrated a strong positive effect on employees’ trust toward the leader and the organization (β = .53, *p* < .001). Leaders’ use of direction-giving language also showed a moderate positive effect on employee trust (β = .20, *p* < .001).

**Table 2.** The Direct, Indirect, and Total Effects of Leaders’ Use of Motivating Language on Employee Trust.

	Meaning-making	Empathetic	Direction-giving
Direct effect on trust	0.11	0.53	0.20
Indirect effect on trust	0.04	0.07	0.07
Total effect on trust	0.15	0.60	0.27

Hypotheses 4 and 5 predicted the positive impacts of leaders’ use of meaning-making, empathetic, and direction-giving language on employees’ psychological need satisfaction for competence and/or relatedness. Results provided support for all of these hypotheses. Specifically, leaders’ use of meaning-making language showed moderate positive effect on employee psychological need satisfaction for relatedness (β = .21, *p* < .001). Leaders’ use of empathetic language showed strong positive effects on both employees’ psychological need satisfaction for competence (β = .28, *p* < .001) and relatedness (β = .26, *p* < .001). Leaders’ use of direction-giving language also showed strong positive effect on employees’ psychological need satisfaction for competence (β = .32, *p* < .001) and relatedness (β = .24, *p* < .001).

Hypotheses 7 and 8 proposed the positive effects of employees’ psychological need satisfaction for competence (H7) and relatedness (H8) on employee trust toward the leader and the organization. Results provided support for both hypotheses. In particular, employee psychological need satisfaction for competence showed small but significant positive effect for employee trust toward the leader and the organization (β = .09, *p* < .01). Employee psychological need satisfaction for relatedness also showed significant positive effects on employee trust toward the leader and the organization (β = .17, *p* < .001).

#### Indirect (mediation) effects

A formal test of indirect effects using a bootstrap procedure (*N* = 5,000 samples) was conducted to test hypothesis 9. Results showed that the indirect effects from leaders’ use of meaning-making (β = .04, *p* = .005 [95% CI: 0.01−0.07]), empathetic (β = .07, *p* = .001 [95% CI: 0.03−0.13]), and direction-giving language (β = .07, *p* = .001 [95% CI: 0.03−0.12]) to employee trust toward the leader and organization via employees’ psychological need satisfaction for competence and relatedness were significant, supporting hypothesis H9.

## Discussion and Conclusions

The purpose of this study was to explore how leadership communication, more specifically, supervisory use of motivating language influences employee trust through satisfying psychological needs during the COVID-19 pandemic. The findings of the study are further discussed below followed by implications of the findings for internal communication scholars, practitioners, and organizational leaders.

### MLT and Employee Trust

Leadership communication has been seen as a powerful catalyst for engendering and fostering employee trust (Mayfield & Mayfield, 2002). While leaders often utilize a myriad of communication strategies and tactics when interacting with their followers, this study focuses on leadership’s use of motivating language and examined how it affects employee trust in both the leader and the organization. The results revealed that all three types of leaders’ motivating language showed strong positive effects on employee trust in leaders and the organization during the COVID-19 pandemic. In particular, when supervisors used meaning-making language to reinforce the culture, values, and purpose of the organization, empathetic language to show concern, care, and appreciation, and direction-giving language to clarify tasks and expectations, employees tended to report a higher level of trust in the organization and the leader.

These findings are not surprising. First, to survive the pandemic, meaning-making language has proven to be efficient in keeping people motivated and resilient in coping with the current realities (Lee, 2020). Either through formal talk or casual conversations, meaning-making messages from leaders can assist employees in achieving value congruence with the organization (Mayfield et al., 2015). According to Edwards and Cable (2009), regardless of the target of the trust, value congruence with the organization could be essential for employee trust to occur. Consistent with prior studies (Gilbert & Tang, 1998; Seppänen et al., 2014), the present study revealed that supervisor use of meaning-making language is necessary for establishing and reinforcing employee trust toward the leader and the organization.

Second, previous research has suggested that leaders who use direction-giving language are viewed as competent in clarifying tasks and goals, which in turn, can foster trust (Hackman, 2002). The role of direction-giving language in reviving the foundation of employee trust becomes even more critical during organizational distress, as setting realistic expectations and providing clear direction can reduce ambiguity and uncertainty (Mayfield & Mayfield, 2002). When employees obtain adequate information and clear feedback from leaders, a belief that their leader is reliable, competent, and honest arises (Ling & Guo, 2020). Therefore, leaders who engage in open, directive, and clear communication may induce higher subordinate trust.

Additionally, employees can develop a sense of trust from expressed consideration and empathy by leadership (Sullivan, 1988). Such emotional support, involving empathy, care, and encouragement can be especially instrumental in aiding employees to cope with uncertainties such as health and mental challenges during the pandemic. Indeed, the pandemic not only disrupts the broad workforce physically and financially, but emotionally as well (Restubog et al., 2020). Supervisors who demonstrate concerns for subordinate well-being can become a source of relief from employee emotional exhaustion (Harms et al., 2017), thereby fostering trust in leaders and organizations. The fact that empathetic language showed the strongest effects among the three types of language on employee trust is evidence of the crucial role of leader communication of emotional support during such challenging times.

### The Underlying Mechanism: Employees’ Psychological Need Satisfaction

Another major finding of this study was that leader use of motivating language (i.e., meaning-making, empathetic, and direction-giving language) had significant positive impact on satisfying employee psychological need for competence and relatedness during the pandemic, which in turn cultivated employee trust toward leadership and the organization. First, leader use of meaning making language that shares the vision, values, and culture of the organization helps employees understand the meaningfulness of their work and feel connected with their organization (Mayfield & Mayfield, 2017). Moreover, meaning-making language serves as a compass during the pandemic in aligning employee personal goals with a higher purpose of the organization, thus creating a sense of meaningfulness among employees, and satisfying their psychological need for relatedness. Second, leader use of empathetic language played an important role in fulfilling employee psychological need for competence as empathetic language in the form of praise and encouragement can increase employee feelings of self-efficacy in dealing with unexpected changes and challenging tasks during the pandemic (Baard et al., 2004; Mayfield & Mayfield, 2018). Moreover, when leaders show authentic care and take employee input into consideration, it can reduce anxiety and cultivate a stronger sense of belongingness to the organization among employees during the pandemic, thus satisfying their psychological need for relatedness (Deci et al., 2001; Weinstein & Ryan, 2010). Third, leader use of direction-giving language provides a clear sense of direction and helps employees reduce their uncertainty and enhance their feeling of self-efficacy (Binyamin & Brender-Ilan, 2018; Mayfield & Mayfield, 2018). Also, receiving timely and clear direction and guidance allows employees to have frequent interactions with their leaders, which may further reduce feelings of isolation and satisfy their need for relatedness during the pandemic.

This study also demonstrates that fulfilling employee psychological need for competence and relatedness can enhance trust toward the leader and the organization during the pandemic. According to the self-determination theory, satisfying the psychological needs of employees can enhance self-growth and mental well-being at work (Deci & Ryan, 2000; Lynch et al., 2005), both of which are beneficial to developing a trusting relationship with the leader and organization. Specifically, previous research has found that when psychological need for relatedness is fulfilled, employees are more willing to cultivate a positive and long-lasting relationship with the organization (Eisenberger et al., 2010; Tao et al., 2018). This study further verified this link in the context of a pandemic and demonstrated that feelings of connection can significantly enhance employee trust towards leadership and the organization, especially considering that employee need for relatedness is more evident than ever during turbulent times (Charoensukmongkol & Phungsoonthorn, 2020). Also, it is logical that if employee psychological need for competence is met at work, they will feel more confident in performing their job effectively and be more likely to connect that accomplishment with support gained from the organization (Tao et al., 2018). Moreover, it is important to note that satisfying employee psychological need for relatedness exerted stronger impact on employee trust compared to satisfaction of competence.

Furthermore, this study explained how leaders’ use of motivating language influenced employee trust by revealing the mediating role of employee psychological need satisfaction. Specifically, if an employee’s psychological need for competence and relatedness is fulfilled via leadership’s effective use of meaning-making, empathetic, and direction-giving language during the pandemic, the employee is likely to believe that their leaders can be relied upon and are professional in leading the team through difficult times (Deci et al., 2001), which ultimately furthers employee trust. In addition, although previous research has shown positive associations between leader use of motivating language and employee relationships with the organization (Tao et al., 2018; Van den Broeck et al., 2016), this study demonstrated the more salient impact of leaders’ use of empathetic language in comparison to direction-giving and meaning-making language on employee trust in the leader and the organization during the pandemic.

### Theoretical and Practical Implications

The findings of this study provide significant implications for public relations, and management communication scholars, practitioners, and organizational leaders. Theoretically, this study contributes to the growing body of literature on leadership communication by demonstrating the impact of supervisory communication on employee trust during organizational turbulent times such as in the midst of an unprecedented pandemic. While numerous studies have documented the impact of supervisory leadership on employee trust, research that particularly focuses on the role of leadership *communication* in fostering trust in the leader and the organization has been limited, even less in a unique public health crisis context. Through the lens of motivating language theory, the study showcased how leadership’s verbal communication with followers affects the employee’s trusting attitude toward the leader and the organization during turbulent times. While a crisis like COVID-19 is a self-evident threat, it provides an opportunity for leaders to reflect on what communication practices can help retain employee trust through a crisis (Stoller, 2020).

In addition, this study adapted the scale of motivating language to fit the COVID-19 context, thus expanding the explanatory power of MLT to the pandemic leadership communication context and providing scholars insights on how to adapt the original MLT scale for a specific situation. Furthermore, this study conceptualized employee trust by incorporating both leadership and organizational-level trust. It also revealed how meeting employee basic psychological needs via effective leadership communication nurtures employee trust, highlighting the importance of an employee-centered approach in internal communication. Last but not least, with a particular focus on a public health crisis context, that is, during the COVID-19 pandemic, the study also advances theoretical understanding of organizational disaster communication with internal stakeholders particularly pertaining to maintaining employee trust. Such knowledge is critical because employee trust serves as the key factor in building resilience during a widespread disaster (Longstaff & Yang, 2008).

From a strategic standpoint, this study provides implications for organizational leaders at different levels of the organization on how to enhance leadership communication, satisfy employee psychological needs, and consequently cultivate employee trust during the widespread economic downturn. First and foremost, leaders need to communicate proactively to employees facing series of pandemic-induced changes and challenges for the workforce such as social distancing and loneliness, stress, health and well-being (Kniffin et al., 2021). In particular, a versatile strategic communication model that includes the use of meaning-making, empathetic, and direction-giving language should guide leadership communication practice which in turn will generate positive employee outcomes (Sullivan, 1988). In other words, during the pandemic, leaders are expected to give clear direction and sufficient information to help followers understand jobs and tasks. Working remotely through virtual team can hardly resemble the richness of face-to-face communication, and therefore it is important for leaders to clearly state their expectations, directions, and vision to assist employees to work efficiently (Kniffin et al., 2021).

Beyond that, leaders should communicate in a way that conveys compassion, empathy, respect, concern, and gratitude to their followers. The long-lasting pandemic has caused widespread emotional distress, anxiety, and boredom, so leaders need to be equipped with emotional intelligence, recognize employees’ emotional needs and communicate timely and sufficient emotional support (Dirani et al., 2020). More importantly, leaders can further motivate and inspire employees by sharing stories of mental models and explaining the organizational culture, norms, and behaviors. In times of pandemic-related loss and life threats, the meaning-making process is important because it helps people make sense of loss, rebuild shared beliefs, reduce self-blame and guilt, and maintain hope for a positive outlook (Walsh, 2020).

In addition, leaders should recognize the importance of employee psychological needs especially during COVID-19 distress. As shown in this study, leaders who clarify work expectations, express support, and concern, and empathize organizational values, meanings and purpose can increase employee sense of self-efficacy and connectedness, thereby making this calamitous time less overwhelming for employees and ultimately enhancing employee trust.

### Limitations and Directions for Future Research

This study encountered several limitations that can be addressed in future research. First, this study employed a quantitative survey to examine the linkages between supervisors’ use of motivating language, employees’ psychological need satisfaction, and employee trust. To gain an in-depth understanding of how supervisory leadership communication influences employee trust during the pandemic, future studies can utilize qualitative approaches such as interviews, case studies, and focus groups. Future research can also focus on a single type of organization or a particular industry (e.g., healthcare, or other essential businesses), or a specific group of employees such as front-line workers during the pandemic to offer more context-specific insights regarding leadership communication during the pandemic. Additionally, the cross-sectional survey design with self-reported data may be subject to common method variance (CMV). Although the use of Harman’s single factor test and the unmeasured latent method factor technique (Fuller et al., 2016; Richardson et al., 2009) did not reveal common source bias in our data, we suggest future research adopt a mixed-method approach or include two or more information sources to rule out any potential CMV influence. Relatedly, although SEM analysis is an advantageous statistical tool in testing priori conceptual models derived from the literature (Kline, 2011), it is limited in establishing true causal relationships. Therefore, we suggest future researchers use experimental and longitudinal designs to further examine the order of effects in our model. Furthermore, this study focused on exploring employee psychological need for competence and relatedness, which are identified as salient mental and emotional needs of employees during the pandemic. Finally, this study focused on supervisory use of motivating language in the U.S. Since the COVID-19 pandemic is a global disease outbreak, it is worthy of examining how the effectiveness of leaders’ use of motivating language may be influenced by national culture and socioeconomic contexts.

## Appendix: Measures of Key Constructs

### Meaning-Making Language

My direct manager/supervisor. . .

(1) Shares stories of mental models during the pandemic.
(2) Tells stories about how employees are working hard during the pandemic.
(3) Talks about vision, values, or culture of the organization during the pandemic.
(4) Connects the work that I do to a higher purpose during the pandemic.
(5) Talks about meaningfulness of my work during the pandemic. (Source: Mayfield & Mayfield, 2017)

### Empathetic Language

My direct manager/supervisor. . .

(1) Gives me praise for my good work during the pandemic.
(2) Shows encouragement of my work efforts during the pandemic.
(3) Shows concern about my health, safety, or wellbeing during the pandemic.
(4) Expresses his/her support for me during the pandemic.
(5) Shows sensitivity for my needs during the pandemic.
(6) Shows trust in me during the pandemic.
(7) Expresses appreciation for my work during the pandemic.
(8) Shows sympathy on the personal difficulties that I (may) face during the pandemic.
(9) Shows understanding for what I’m going through during the pandemic. (Source: Mayfield & Mayfield, 2017)

### Direction Giving Language

My direct manager/supervisor. . .

(1) Gives me clear directions of what needs to be done in my work during the pandemic.
(2) Provides me with easily understandable instructions about my work during the pandemic.
(3) Offers me specific information on how my work will be evaluated during the pandemic.
(4) Gives me useful explanations of how my job is impacted by the pandemic.
(5) Provides me with helpful information about forthcoming changes affecting my work due to the pandemic.
(6) Shares news with me about the organizations’ functions, operations and financial status during the pandemic (Source: Mayfield & Mayfield, 2017)

### Psychological Need Satisfaction for Competence

(1) I feel adequately prepared to perform my job during the pandemic.
(2) I feel competent at my job during the pandemic.
(3) I have the feeling that I can accomplish difficult tasks at work during the pandemic.
(4) I have the skill to perform my job well during the pandemic.
(5) I can complete my tasks effectively most of the time during the pandemic.
(6) I feel confident about my ability to do my job during the pandemic. (Source: Van den Broeck et al., 2010)

### Psychological Need Satisfaction for Relatedness

(1) I feel connected with other people in my organization during the pandemic.
(2) I feel part of a group at my organization during the pandemic.
(3) I can talk with people in my organization about things that matter to me during the pandemic.
(4) I feel isolated from other people in my organization during the pandemic. (reverse coded)
(5) I feel a sense of communion at work during the pandemic.
(6) I feel cared for by others in my organization during the pandemic (Source: Van den Broeck et al., 2010)

### Employee Trust

#### Trust in leader

(1) I can freely share my ideas, feelings, and hopes with my supervisor during the pandemic.
(2) I can talk freely to my supervisor about difficulties I am having at work during the pandemic and know that (s)he will want to listen.
(3) If I shared my problems with my supervisor during the pandemic, I know (s)he would respond constructively and caringly.
(4) I would have to say that my supervisory has made considerable emotional investments in our working relationship during the pandemic.
(5) My supervisor approaches his/her job with professionalism and dedication during the pandemic.
(6) Given my supervisor’s track record, I see no reason to doubt his/her competence and preparation for the job during the pandemic.
(7) I can rely on my supervisor not to make my job more difficult by careless work during the pandemic (Source: McAllister, 1995)

#### Trust in the organization

(1) My organization treats people like me fairly and justly during the pandemic.
(2) Whenever my organization makes an important decision during the pandemic, I know it will be concerned about people like me.
(3) My organization can be relied on to keep its promises during the pandemic.
(4) I believe that my organization takes the opinions of people like me into account when making decisions during the pandemic.
(5) I feel very confident about my organization’s skills during the pandemic.
 My organization has the ability to accomplish what it says it will do during the pandemic (Source: Hon & Grunig, 1999)

## Notes

1. Considering the sample size of the sample, one-level SEM analysis was utilized.
